# Use of bone marrow cells (BMCS) added to Platelet-Rich Plasma (PRP) for treatment of bone degenerative processes in JIA patients: a case report

**DOI:** 10.1186/1546-0096-9-S1-P188

**Published:** 2011-09-14

**Authors:** MG Alpigiani, P Salvati, M Muraca, S Callegari, G Tripodi, R Lorini, MB Michelis, S Boero

**Affiliations:** 1 Department of Pediatry, Gaslini Institute, Genoa, Italy; 2 Service of Immuno-Hematology and Transfusion Medicine, Gaslini Institute, Genoa, Italy; 3 Department of Orthopedics, Gaslini Institute, Genoa, Italy

## Introduction

In Regenerative Medicine one or more regenerative factors can be applied inside a cartilagine or bone defect to obtain a more rapid and complete healing. Bone Marrow Cells (BMC) added to Platelet-Rich Plasma (PRP) contain stromal cells which can differentiate in osteoclasts and osteoblasts and can be able to form osteogenic tissue and to repair bone defects secondary to degenerative processes.

## Case report

We report on a 15-year-old boy, followed at our Department, affected by JIA since he was 2 years old. He presented a systemic form, evolved into a polyarticular form, treated with steroids and immunosuppressor drugs. The patient had a good response to treatment with Enbrel, which he is still taking. In January 2009, he presented right hip pain and functional limitation. In July 2009 he underwent MRI of the hip joints which showed osteonecrosis in chondral/subchondral regions at the superior-external convexity of the right femoral head. We recommended deambulation with crutches and no weight bearing. Because of the persistence of joint symptoms, in July we implanted BMC plus PRP in the osteonecrotic region with improvement of pain and mobilization. In October 2010, he presented left hip pain. MRI showed focal osteonecrosis in subchodral region of left femoral head convexity. For this reason, we made a second BMC plus PRP implantation in the left hip. At present, patient walks autonomously, without joint pain and with improved hip movements; he is still on Enbrel and NSAIDs.

## Conclusions

To the best of our knowledge, there are no literature data on the use of BMC plus PRP in pediatric patients affected by JIA. Considering the obvious limitations of our single case report, we observed a good short-term outcome. Therefore, follow-up is essential to check if BMC plus PRP implantation represents only a palliative care to delay surgical treatment or if it is a valid alternative to traditional orthopedic surgery.

